# Prediction of Walnut Deterioration Using Kernel Oxidative Stability

**DOI:** 10.3390/foods9091207

**Published:** 2020-09-01

**Authors:** Filipa Simoes Grilo, Yanisa Srisaard, Selina C. Wang

**Affiliations:** 1 Department of Food Science and Technology, University of California, Davis, CA 95616, USA; fsgrilo@ucdavis.edu (F.S.G.); ysrisaard@ucdavis.edu (Y.S.); 2 Olive Center, Robert Mondavi Institute for Wine and Food Science, University of California, Davis, CA 95616, USA

**Keywords:** peroxide value, storage, Rancimat, hexanal, rancidity, sensory evaluation

## Abstract

Monitoring walnut oxidation is essential to control walnut quality during storage. An accelerated oxidation method for differentiating the oxidative stability index (OSI) of walnut kernels was examined and the effects of instrument operational parameters such as temperature and airflow were evaluated. Four cultivars, Chandler, Solano, Durham, and Howard were analyzed at 110, 120, and 130 °C with 15, 20, and 25 L h^−1^ airflow. Analysis using 110 °C with 25 L h^−1^ yielded the lowest coefficients of variance (4.4) than other operational parameters; analysis using the same temperature at lower airflow, 15 L h^−1^, yield the highest coefficient of variance (10.5). Kernel OSI values were independent of airflow, however, dependence of temperature coefficient and Q_10_ were demonstrated. The results from selected parameters were correlated with fat and moisture content, peroxide value, UV absorbances, oil oxidative stability, hexanal, and rancidity to establish the relationships between OSI values and quality changes during storage. Using 0.5 g of ground kernels, at 110 °C with 25 L h^−1^ airflow gave a lower coefficient of variance and higher correlation with kernel quality and oxidative markers comparing to other combinations of operating parameters.

## 1. Introduction

Walnuts are known to contain not only high levels of triglycerides, but also high proportions of unsaturated fatty acids (oleic acid, linoleic, and linolenic acids), which have long been considered as relevant health-promoting components. Unsaturated fatty acids can be easily oxidized in the presence of oxygen, light, moisture, and heat [1]. Fatty acid oxidation is initiated by reactive oxygen species, which lead to the formation of various primary and secondary products [2]. These products result in the modification of major quality characteristics such as color, flavor, aroma, and nutritional value, affecting the suitability of a food product [1].

Fatty acid compositions of walnuts contribute to a shorter shelf life compared to other nuts, which presents challenges during storage and limits the usage as a snack mix product [1]. Since lipid oxidation occur slowly at room temperature, accelerated methods can be applied to estimate the oxidative stability of a product in a more rapid time frame, particularly if the temperature is exponentially related to the rate of reaction [3]. Common accelerated oxidation methods include the active oxygen method, Schaal Oven method, and Rancimat method [4]. Rancimat method observes the production of volatile organic compounds while the other two methods look at peroxide formation during oxidation. Rancimat instrument is simple to use and has good reproducibility [5,6].

Rancimat method was developed by Hadorn and Zurcher (1974) [7] to measure oxidative stability and has been widely used to determine lipid oxidation and predict the shelf life of oils and fats [8]. This method has been vastly used to study the effects of antioxidants, edible coatings, and storage conditions of nut oils [9,10,11]. However, measuring oxidative stability through an accelerated method can lead to an over or underprediction of the actual shelf life due to different mechanisms of peroxidation that occur at room temperature [4,12]. Consequently, the study and use of the proper operational parameters is essential to avoid misleading results in accelerated oxidation methods. Nut oxidation depends on fat content, moisture, fatty acid composition, and antioxidant content; these characteristics may vary significantly with cultivar and year of production [13,14,15].

The use of Rancimat for high-fat solid-foods is not commonly used [16], and there are no studies on the effect of temperature and flow parameters on oxidative stability index (OSI) values for walnut kernels. The aim of this work was to study the capability of this accelerated oxidation method in differentiating oxidative stability of walnut kernels and evaluate different operating parameters of the instrument. Four walnut cultivars were analyzed using Rancimat at three airflow rates and three temperatures to allow the identification of the operational parameters with the least variance coefficient to measure oxidative stability. To study the relationships between oxidation levels and OSI values of walnut sample, we also measured kernel OSI, quality parameters, and oxidative markers over six months of storage. Findings from this study would help monitor the oxidative quality of walnut kernels without the need for oil extraction prior to analysis.

## 2. Materials and Methods

### 2.1. Samples Preparation

Walnut fruits (Juglans regia—Chandler, Solano, Durham, Howard) were collected from the University of California walnut breeding program, Oakland, California, USA. After cleaning, the fruits were dried at 30 ± 2 °C in a day using a commercial air dryer. For each cultivar, 10 kg of dried and shelled walnuts were stored at 0 °C, and hand-cracked before each analysis.

### 2.2. Oxidative Stability Index

The oxidative stability index (OSI) was measured using a Rancimat apparatus 617 (Metrohm AG, Herisau, Switzerland). Samples were placed into test tubes, and air was bubbled inside at a controlled flow in block heated at a controlled temperature. Effluent air containing volatile organic acids from the samples was collected in a measuring vessel containing double distilled water (50 mL). The conductivity of the water was automatically measured as oxidation proceeded. For kernel oxidative stability, 0.5 kg of walnuts were cracked and grounded using a Cuisinart Mini-Prep Plus^®^ processor (East Windsor, NJ, USA), model DLC-2A. A sample from those ground walnuts kernels (0.5 g) was weighed into test tubes and oxidized under the testing conditions. Filtered, cleaned, and dried air bubble through the kernel samples at the rates of 15, 20, and 25 L h^−1^. OSI is the time (in hours) taken until a sharp increase in conductivity occurs, which is determined by the intersection of the baseline with the tangent to the conductivity curve [17]. The OSIs of the samples were recorded at 110, 120, and 130 °C. In each run, eight samples were randomized on their position in the heating block and analyzed simultaneously. For oil oxidative stability, oil samples (3 g) were weighed into test tubes and oxidized under the testing conditions of 110 °C with 20 L h^−1^ of airflow following the instrument manufacture recommendations.

### 2.3. Storage Conditions

Samples from Chandler and Howard cultivars were stored at 25 °C and 60% Rh for 6 months. Samples were collected before storage, at four months of storage, and after six months of storage. Kernels and extracted oil were used to obtain quality parameters to compare with different Rancimat operational parameters.

### 2.4. Moisture Content

Previously grounded kernel samples (40.0 ± 0.1 g) were weighed in a 600 mL beaker and placed in the oven at 105 °C until constant weight. The beaker was then transferred to a desiccator and the weight of the dry paste registered at room temperature. Each sample was analyzed in triplicates.

### 2.5. Fat Content

The total fat content of the previously dried ground walnuts was obtained by Soxhlet standard extraction mode of the Buchi extraction system (E800, Buchi Labortechnik AG, Switzerland) using n-hexane and 50 extraction cycles with 30 min rinsing. Each sample was analyzed in triplicates.

### 2.6. Walnut Oil Extraction

Oil extraction was carried out with a screw press, KK oil Prince F universal (Reut, Germany), with a 7 mm restriction die and a screw speed of 20 rpm. The screw press was first run for 10 min without seed material but with heating means (30 °C), an electrical resistance-heating ring, attached around the press barrel to raise the screw-press barrel temperature to the desired temperature. The running temperature was checked with a digital thermometer. Each treatment was done in triplicate; all press devices were cleaned and dried after each run.

### 2.7. Quality Parameters

Peroxide value, and UV absorbances (K_232_, K_270_) values were determined according to standard methods of American Oil Chemistry Society, 2009, Cd 8b-90(09), and Ch 5-91(09), respectively.

### 2.8. Hexanal Concentration in Kernels

Hexanal was extracted using solid-phase microextraction (SPME). Samples of walnut grounded (3 g ± 0.1 g), spiked with 4-methyl-2-pentanol as internal standard (2.5 mg/kg), was weighed into a 20 mL glass vial and sealed with a PTFE/silicone septum (Agilent Technologies, Palo Alto, CA, USA). After 10 min in agitation mode, at 40 °C, a solid-phase microextraction (SPME) fiber (DVB/CAR/PDMS, Sigma-Aldrich, St. Louis, MO, USA) was exposed to the sample headspace for 40 min for volatile extraction. The hexanal analysis was performed in triplicate using a GC system (Agilent Technologies, Palo Alto, CA, USA) that comprise an autosampler (Agilent PAL RSI 85), a gas chromatograph (GC Agilent 7820A), and a mass spectrometer (Agilent 5977B) that included an electron impact source and a quadrupole analyzer. A Supelcowax 10 (30 m × 0.25 mm × 0.25 μm, Sigma-Aldrich, St. Louis, MO, USA) was used for compounds separation. Helium was used as carrier gas at a flow rate of 1 mL/min. GC oven temperature started at 40 °C and ramped at 3 °C/min after 10 min to the final temperature of 200 °C. The data were recorded and analyzed using Agilent MassHunter Qualitative Analysis. Hexanal was identified using the NIST 08 Mass Spectral Library and via comparison with the standard (Sigma-Aldrich, St. Louis, MO, USA).

### 2.9. Sensory Analysis

Sensory analysis of walnut kernels was performed on samples at the initial stage and after four and six months of storage. Descriptive Analysis was performed by trained panelists using reference samples [18,19]. Ten trained descriptive panelists (2 M; 8 F age range 23–68 years) were used to detect and rate intensity of rancidity attribute in kernels. Judges were selected on their ability to recognize rancidity. Judges were trained for the descriptive analysis procedure in five one-hour training sessions. The training involved judges learning to recognize rancidity in the kernels, distinguish samples, and use a 10-point intensity scale. These were done using walnut oil storage for 36 months (at 23 °C under artificial light) as a rancidity standard. After training, judges were given the samples to evaluate in triplicate and randomly distributed between evaluation sessions and judges. Each sample contained two walnut kernels prepared in glasses and covered with a lid approximately two hours before evaluation. Four samples were evaluated in each session. Samples were evaluated in individual sensory booths under red light and identified by a three-digit numerical codes to minimize bias. Judges could re-taste the kernels as often as necessary and had to rinse with water and eat a grape between sample tastings. Each session lasted approximately 20 min.

### 2.10. Shelf-Life Prediction, Temperature Coefficient, and Q_10_ Number Calculation

The logarithms of OSIs versus elevated temperatures (110, 120, and 130 °C) were plotted, and lines were fitted to the data. The equation for each line was also determined, and the slopes of the lines generated the temperature coefficients (T_coeff_). The shelf-lives as the kernel OSIs at 22 °C (OSI_22_) were calculated from the corresponding equations. Q_10_ numbers were calculated as the OSI at T/OSI time at T + 10 °C. The coefficient of variance (CV) of the different treatments was calculated by CV = (SD/average) × 10 where SD is the standard deviation of each treatment applied.

### 2.11. Data Analysis

Quality parameters and GC-MS analysis were performed in triplicate per replicate of oil extraction per cultivar (*n* = 36). Each oxidative stability treatments (temperature and air flow) were performed on all eight Rancimat positions per cultivar (*n* = 288). For each cultivar, at harvest and two storage times (four and six months), correlations were performed using four different treatments on all Rancimat positions (*n* = 288). All data were tested by analysis of variance (ANOVA) using SYSTAT procedures (Systat Software Inc., Chicago, IL, USA). Significant storage effects for each cultivar were followed by Tukey’s multiple comparison test at *p* ≤ 0.05. Correlation analysis was performed using Pearson’s test.

## 3. Results and Discussion

### 3.1. Effect of Operational Parameters on Kernel OSI Measurements

Results of oxidative stability measured in the walnut kernels at three different temperatures and three different airflow rates are shown in Table 1. All temperatures affect the oxidation stability of the kernel significantly (*p* < 0.0001). The induction time was about halved with each 10 °C increase in temperature. Measurements at 110 °C were significantly higher than measurements at 120 °C and subsequently at 130 °C, with an average of 13.9, 5.3 and 2.6 h, respectively. Differences between airflow rates were significant only at 110 °C with 20 L h^−1^, giving the highest oxidative stability values. Contrary to our results, studies in soybean oil, demonstrated that airflow above 15 L h^–1^ significantly affect the OSI [20]. In a fluid matrix, it is more difficult to reach the equilibrium at higher airflows. Our results showed that with small sample size, the equilibrium was reached, giving no significant differences between airflow rates.

As the accelerated method was conducted on the same samples with different operational parameters, direct comparisons of the absolute value of induction times could not provide insightful information. Instead, the coefficients of variance (CV) were calculated to distinguish operational parameters effects on OSI and determine the best parameter combination to evaluate walnut kernels (Table 1). At lower temperature (110 °C), airflow showed to be decisive for the reproducibility of the method, with 15 L h^−1^ giving the highest and 25 L h^−1^ the lowest CV. The highest temperature (130 °C) consistently yielded higher CV for all flow rates studied (7.6–8.1). By excluding the lowest airflow (15 L h^−1^) and the highest temperature (130 °C), we narrowed down the operational parameters for further investigations.

### 3.2. Shelf-Life Prediction, and Temperature Coefficient, and Q_10_ Number Calculation

Choosing the most suitable operational parameters for foods with different composition and matrix is important for the application, accuracy, and reproducibility of accelerated oxidation methods. In addition, it was previously demonstrated that susceptibility to oxidations in different levels of n-3 PUFA-rich oils such as algae and fish using Rancimat method and chemometric analysis can be accomplished [3]. Thus, a reliable accelerated oxidation method for walnut kernels would be useful and valuable information to distinguish the susceptibility to oxidations.

A linear regression between the logarithm of kernel OSI at three different temperatures and temperature coefficients were calculated to predict shelf-life using the accelerated method (Table 2).

Significant linearity was found for each airflow, with R^2^ of 0.997, 0.989, and 0.988, respectively. The temperature coefficient (T_coeff_) represents the factor by which the rate (R) of a reaction increases for every 10-degree rise in the temperature (T), which in our case indicates the slope of our linear relations. T_coeff_ showed similar values from 15 and 20 L h^−1^ (−3.7 and −3.9, respectively) but lower values at the highest airflow (−3.3). The decrease rate of OSI with a 10 °C increase in temperature from this study is much higher than 2, the rate found in oils and edible fats in previous studies [6,20]. The low curve slope at an airflow 25 L h^−1^ determined a lower OSI_22_ and Q_10_. OSI_22_ indicates the oxidative stability of samples if measured at 22 °C. This value represents an estimation of the time required for the samples to reach oxidative stability if exposed at 22 °C of storage, which is the temperature of exposure of most products in-store shelves. The highest value was found for airflow at 15 L h^−1^, and the most conservative shelf-life prediction was given by airflow at 25 L h^−1^. These results suggest that the airflow influences the shelf-life prediction for walnut kernels using the Rancimat method. Q_10_ is the factor by which the rate increases when the temperature is raised by ten degrees. In this study, an airflow of 15, 20, and 25 L h^−1^ had Q_10_ of 2.4, 2.5, and 2.2, respectively. These results are in accordance with previous studies on oil and ghee using the same temperature ranges [5,21]. When increasing the temperature of analysis by 10 °C, accelerated oxidative reactions and OSI are halved. The possibility to decrease OSI by increasing temperature may give decrease time of analysis which may be important as OSI in kernels (Table 1) is much higher than the OSI in oil [22]. This method is simple and does not require a trained scientist so it can be done easily in-house at processing facility, an advantage compared to other oxidative methods commonly used in kernels, such PV, volatile compounds or sensory evaluations.

### 3.3. Relations between Kernel OSI and Quality of Kernel and Extracted Oil

Many methodologies have been developed and implemented for the determination of both primary and secondary oxidation products, including sensory and chemical analysis, such as peroxide value (PV), UV absorbances and volatile compounds [23]. PV, UV absorption and sensory methods determine the oxidation of a sample in real-time, while oxidative stability (Rancimat induction time) indicate oxidative susceptibility and stability. We evaluated the OSI operational parameters on walnut kernels by establishing the relation between traditional methods for measuring primary and secondary oxidation products and kernel OSI. Since we observed small coefficient of variance when analyses were conducted at 110 and 120 °C and 20 and 25 L h^−1^ (Table 1), these parameters were selected to further study the relation of the kernel OSI and other kernel quality and oxidative markers currently used by industry and researchers. Samples at harvest, four and six months after harvest were used for correlation analysis. While correlations do not prove the equivalence between the results, it provides a measure of the strength of a relationship between two methods. Correlations between kernel OSI and kernel moisture, fat content, hexanal and rancidity, and oil OSI, PV, UV absorbances at 232 (K_232_), and 268 (K_268_), were studied (Table 3). Moisture had no significant linear correlation with kernel oxidative stability using any of the studied parameters. Temperature at 120 °C and Airflow 20 L h^−1^ did not show significant values of Pearson correlations with rancidity. This lack of significant relationship may be due to a lower range of values obtained in the Rancimat, using those operational parameters (2.7 to 6.8 h), compared to the range of rancidity (0 to 6.7) detected in the samples. On the other hand, OSI measured at 110 °C with 20 and 25 L h^−1^, and 120 °C with 25 L h^−1^ had a high and significant negative correlation (−0.7, −0.8 and −0.7, respectively) with rancidity detected in the samples. A higher OSI corresponded to less rancidity perception. The Pearson correlation coefficient was higher (*r* > 0.7), mostly in the measurements at 110 °C with both 20 and 25 L h^−1^ airflows. A study on potato chips using 110 °C and 20 L h^−1^, concluded that using those operational parameters, OSI could be measured directly from the oil-based food samples rather than from the extracted oil [24]. However, that study did not include a comparison of operational parameters.

### 3.4. Relations between Kernel OSI and Quality of Kernel and Extracted Oil during Storage

Lipid oxidation at low and high temperatures may go through different reaction pathways, depending on the reactivity of metal ions and antioxidants at different temperatures [20,25]. Thus, it is important to mention that Rancimat methods may lead to either overprediction or underprediction of the actual shelf life depending on the type of sample tested [5]. In order to use Rancimat as a shelf-life prediction method, it is a must to compare the OSI with quality and oxidative markers of samples that have been stored under a more normal condition. Therefore, to further investigate the application of this method and operational parameters to walnut kernels, the OSI of the samples were measured after being stored at 25 °C for four and six months. The results were compared with other quality and oxidative parameters at the beginning and after four and six months of storage (Table 4). After four and six months of storage, moisture and fat content of the samples, demonstrated no linear correlation with OSI for all operational parameters. PV, UV absorbances, hexanal and sensory rancidity demonstrated negative and significant correlations after four and six months of storage for operational parameters using 110 °C (*r* < −0.5; *p* < 0.05). Operational parameters using 120 °C, had the smaller range of OSI values (6.8 to 2.7 h using 20 L h^−1^; 6.2 to 3.2 h using 25 L h^−1^) compared with analysis at 110 °C (17.1 to 6.1 h using 20 L h^−1^; 14.6 to 5.6 h using 25 L h^−1^). The narrow range of OSI for operational parameters using 120 °C resulted in no significant correlations and low coefficients comparing to the range of other oxidative parameters that changed during storage. Higher temperatures shorten analysis time and distinguish lower oxidation stages from higher oxidation stages. However, it also decreases the resolution of the method, due to the narrower OSI ranges and correlations with other quality and oxidative parameters are possibly lost.

Results showed that operational parameters using 110 °C with 25 L h^−1^ airflow gave the best proximity to values obtained from other methods used to measure walnut oxidation (*r* > 0.6), such as PV, hexanal and sensory evaluation of rancidity. Measuring kernel OSI at 110 °C with 25 L h^−1^ also maintained the linear correlation when samples had different oxidation levels since the Pearson correlation coefficients were also significant among samples at different storage times. It is important to emphasize that measurements at 110 °C with 25 L h^−1^ of airflow also gave the lowest variance among replicates (Table 1).

Rancimat method has been widely used in research and industry for oil analysis and strong correlations were previously described between chemical composition and OSI of the samples [26]. In a previous study, no significant correlation was found between shelf life at room temperature and Rancimat induction time; the authors discouraged the use of this method to predict shelf life for olive oil [25]. However, the authors based their conclusions on the differences between oxidation changes at room temperature compared to accelerated oxidations changes in the samples from the Rancimat. Nowadays, OSI is commonly used as a part of prediction models to evaluate oils shelf life, not a direct comparison to actual storage shelf life [8].

Oil extraction from the walnut kernels has an impact on oil characteristics that may influence oil oxidation which give misleading information on the susceptibility of kernels to oxidation [27]. Besides oil extraction conditions, the susceptibility of iodine (used for PV analysis) to oxidation in the presence of oxygen can be a further source of variability when analyzing a sample after lipid extraction [28]. In addition, peroxide values do not always give a reliable measure of the oxidation, since peroxides can further degrade in advanced oxidation, so simultaneous measurement of secondary products is appropriate [29]. Therefore, the development of a methodology that predict walnut deterioration using the kernels without prior oil extraction would simplify sample evaluation and decrease unpredictable variations on the sampling process. In addition, Rancimat oxidative stability, contrary to chromatographic methods and sensory analysis, does not require long or meticulous experimental work, an experienced analytical chemist or lengthy the data processing.

Knowing that oxidation processes may be different when using an accelerated method, in this study we focus on establish a relation between the OSI obtained by Rancimat method on kernels and quality parameters of each sample analyzed. Most correlations between oxidative parameters and OSI during storage had a coefficient higher than 0.5 and were significant (*p* < 0.001). Therefore, the results from this study could lead to an application of accelerated oxidation and establishment of acceptable value for walnut kernel stability and shelf life for the industry.

## 4. Conclusions

Measurements done on walnut kernel using Rancimat with temperature at 110 °C and 25 L h^−1^ airflow rate gave the lowest variance and best correlations with parameters important for walnut quality, such as PV and hexanal and rancidity of the kernels. This significant correlation was confirmed by the kernel oxidative stability measured by the same operational parameters (110 °C and 25 L h^−1^) and finding higher values of Pearson coefficient after four and six months of storage.

Advantages of this methods include minimal pre-treatment of samples, small sample size, rapid data processing and no need for specialized personnel. The validation of a rapid and reliable method for oxidative stability determination on walnuts opens new possibility for predicting shelf life of walnut kernels with different fatty acids and antioxidant contents without oil extractions.

## Figures and Tables

**Table 1:** Kernel oxidative stability index (OSI) expressed in hours; and coefficient of variance (CV) of the treatment combinations at different airflows and temperatures.

Flow Rate (L h^−1^)	Temperature (°C)					
	110		120		130	
	OSI	CV	OSI	CV	OSI	CV
15	13.2 ^b^	10.5	5.2 ^c^	8.9	2.4 ^d^	8.1
20	14.9 ^a^	6.9	5.2 ^c^	5.7	2.5 ^d^	8.4
25	13.5 ^b^	4.4	5.4 ^c^	7	2.9 ^d^	7.6

Different letters show significant difference from the interaction of temperature × flow variables at *p* < 0.05 from Tukey test.

**Table 2:** Shelf-life prediction, temperature coefficient and Q_10_ number calculation. Results from the linear relationship between the logarithms of kernel oxidative stability index (OSI) assessed by Rancimat at 110, 120 and 130 °C.

Flow Rate (L h^−1^)	log OSI = A(T) + B			T_coeff_ (×10^–2^)	OSI_22_ (h)	Q_10_
	A ± SE	B ± SE	R^2^			
15	−0.037 ± 0.002	5.181 ± 0.239	0.997	−3.7 ^a^	23,281 ^b^	2.4 ^a^
20	−0.039 ± 0.004	5.414 ± 0.483	0.989	−3.9 ^a^	35,975 ^a^	2.5 ^a^
25	−0.033 ± 0.004	4.783 ± 0.444	0.988	−3.3 ^b^	11,403 ^c^	2.2 ^b^

Standard error (SE); Temperature coefficient from linear slope (T_coeff_); oxidative stability index at 22 °C (OSI_22_); temperature coefficient (Q_10_). Letters show significant differences between flow rate treatments with *p* < 0.05.

**Table 3:** Correlations between kernel oxidative stability index (kernel OSI), quality and oxidative parameters of the walnut kernels and extracted oils.

	Operational Parameters	Moisture	Fat	PV	K_232_	K_268_	Hexanal	Oil OSI	Rancidity
Kernel OSI	110 °C and 20 L h^−1^	0.108	−0.535 *	−0.802 **	−0.71 **	−0.781 **	−0.730 **	0.813 **	−0.730 **
	110 °C and 25 L h^−1^	0.364	−0.487 *	−0.813 **	−0.562 **	−0.710 **	−0.873 **	0.741 **	−0.799 **
	120 °C and 20 L h^−1^	−0.167	−0.486 *	−0.444 **	−0.564 **	−0.617 **	−0.374 **	0.317 *	−0.298
	120 °C and 25 L h^−1^	0.220	−0.609 *	−0.643 **	−0.523 **	−0.618 **	−0.556 **	0.605 **	−0.658 *

(*) means significant with *p* < 0.05; (**) means significant with *p* < 0.001.

**Table 4:** Correlations between kernel oxidative stability (kernel OSI) and quality and oxidative parameters of the walnut kernels and extracted oils during 6 months of storage.

Operational Parameters	Storage (Months)	Moisture	Fat	PV	K_232_	K_268_	Hexanal	Oil OSI	Rancidity
110 °C and 20 L h^−1^	0	−0.772 **	−0.378	−0.604 **	−0.372	−0.533 *	−0.355	0.643 *	−0.973 **
	4	0.855 *	0.094	−0.832 *	−0.896 **	−0.804 *	−0.921 *	0.698 *	−0.877 **
	6	0.054	0.089	−0.817 **	−0.017	−0.519 *	−0.828 **	0.784 **	−0.831 **
110 °C and 25 L h^−1^	0	−0.484 **	−0.432 *	−0.719 **	−0.632 **	−0.604 **	−0.876 **	0.757 *	−0.764 *
	4	0.783 *	0.047	−0.703 *	−0.893 **	−0.625 *	−0.929 **	0.653 *	−0.918 **
	6	−0.457	−0.017	−0.874 **	−0.487	−0.759 **	−0.913 **	0.950 **	−0.921 **
120 °C and 20 L h^−1^	0	−0.774	−0.726 **	−0.648 **	−0.688 **	−0.543 **	−0.553 **	0.369	−0.864 **
	4	−0.216	−0.417	−0.162	−0.351	−0.606 *	−0.280	0.366	−0.355
	6	0.023	0.043	−0.500	−0.291	−0.710 *	−0.542	0.528	−0.546
120 °C and 25 L h^−1^	0	−0.351	−0.528 **	−0.713 **	−0.590 **	−0.365	−0.349	0.266	−0.930 **
	4	0.065	−0.371	0.524	0.052	−0.630	−0.678	0.656	−0.165
	6	0.861 **	−0.660 **	−0.345	0.108	−0.128	−0.189	0.136	−0.143

(*) means significant with *p* < 0.05; (**) means significant with *p* < 0.001.
